# Impact of Wide-Ranging Nanoscale Chemistry on Band Structure at Cu(In, Ga)Se_2_ Grain Boundaries

**DOI:** 10.1038/s41598-017-14215-0

**Published:** 2017-10-26

**Authors:** Adam Stokes, Mowafak Al-Jassim, David Diercks, Amy Clarke, Brian Gorman

**Affiliations:** 1 Colorado School of Mines, Material Science, Golden, CO USA; 2 National Renewable Energy Laboratory, National Center for Photovoltaics, Golden, CO USA

## Abstract

The relative chemistry from grain interiors to grain boundaries help explain why grain boundaries may be beneficial, detrimental or benign towards device performance. 3D Nanoscale chemical analysis extracted from atom probe tomography (APT) (10’s of parts-per-million chemical sensitivity and sub-nanometer spatial resolution) of twenty grain boundaries in a high-efficiency Cu(In, Ga)Se_2_ solar cell shows the matrix and alkali concentrations are wide-ranging. The concentration profiles are then related to band structure which provide a unique insight into grain boundary electrical performance. Fluctuating Cu, In and Ga concentrations result in a wide distribution of potential barriers at the valence band maximum (VBM) (−10 to −160 meV) and the conduction band minimum (CBM) (−20 to −70 meV). Furthermore, Na and K segregation is not correlated to hampering donors, (In, Ga)_Cu_ and V_Se_, contrary to what has been previously reported. In addition, Na and K are predicted to be n-type dopants at grain boundaries. An overall band structure at grain boundaries is presented.

## Introduction

A considerable surge in research has been directed toward introducing renewable energy technologies to today’s energy marketplace. Natural power sources such as wind, biomass, hydro, and solar constitute the most mature and viable renewable energy candidates^1^. The solar industry is currently made up of many different technologies that can be divided into two groups: Si-based wafers (polycrystalline, monocrystalline) and thin films, which currently occupy ~93% and ~7% of the market share, respectively^1^. Chalcopyrite and zinc-blende thin films (CdTe, CZTS, CIGS), along with perovskite technologies, have experienced large gains in record efficiency over the past four years^2^. Perovskite solar cells, although having increased the most in efficiency, have not yet entered the marketplace due to reliability issues^3^. Recent laboratory record-efficiency CdTe and CIGS cells have overcome those of poly-Si (poly-Si occupies 43.9% of global annual production), which may be indicative of a larger market share in the future^1^. Nevertheless, the efficiency gains have largely been empirically based, and a fundamental understanding of causes for the latest improvements is still lacking^4^. Further improvements in scalability, uniformity, and efficiency are likely still accessible. Recent CIGS increases in efficiency have stemmed from adding K into the solar cell absorber^5–7^. Many recent works have concluded that an alkali post-deposition treatment leads to surface modifications responsible for the improved efficiencies^7–11^. However, the role of alkalis at grain boundaries, as well as grain-boundary chemical character in general, still needs to be explored.

The unique self-compensating properties at CIGS surface and grain boundaries may be a significant reason why the material is efficient and cost effective. Interestingly, monocrystalline record efficiencies for CIGS are less than those of polycrystalline CIGS^12^. This goes against intuition, which assumes that a single crystal contains fewer defects than its polycrystalline counterpart, and therefore has longer charge-carrier lifetimes due to less recombination, leading to a higher cell efficiency. Perhaps the reason why poly-Cu(In, Ga)Se_2_ are capable of high-efficiencies is that their grain boundaries are either benign or beneficial. Or, do defects segregate to grain boundaries leaving intra-grain crystallinity purer than monocrystalline material? One theory held for over two decades is that the grain boundaries are naturally Cu-poor, which leads to a lower valence-band maximum (VBM)^13–17^. Because high-efficiency Cu(In, Ga)Se_2_ is a p-type semiconductor, a lowered VBM would be a hole barrier that would block holes from recombining with electrons at defect-rich grain boundaries. Various atom probe tomography (APT) and scanning transmission electron microscopy (STEM) energy dispersive X-ray spectroscopy (EDS)/electron energy loss spectroscopy (EELS) experiments have demonstrated that grain boundaries may be both Cu-poor and In-rich, as well as Cu-rich and In-poor^18–20^. This contribution will show that a wide variety of chemistries exist at grain boundaries and will help to elucidate the type of potential barriers.

Furthermore, Na segregation and more recently Na and K segregation for alkali-incorporated solar cells has been revealed by APT^13,19–23^. However, a minimal number of grain boundaries have been analyzed, and a statistical picture of the chemical variability is needed. The incorporation of Na, either by diffusion from the glass substrate or by post-deposition treatments, has been correlated with large grain sizes and increases in p-type conductivity, resulting in higher efficiency. Na has been theorized to assist in reducing n-type defects, In_Cu_ and/or V_Se_, thereby increasing the p-type conductivity and open-circuit voltage (V_oc_)^24–27^. Here we show no clear relationship of Na (K) to (In, Ga)_Cu_ and V_Se_ defect density at grain boundaries (GBs).

A wide variety of electrical properties at GBs have been reported via Kelvin probe force microscopy (KPFM), electron beam induced current (EBIC), cathodoluminescence (CL), photoluminescence, and EELS. For example, a change in work function from the grain interior to the grain boundary, ranging from +550 meV to −250 meV has been reported^28–32^. A “redshift” was observed at GBs via CL, indicating a donor-acceptor-like transition, presumably (In, Ga)_Cu_ and V_Cu_. Furthermore, a “blue shift” was also observed with excitation at low temperatures, indicating a reduction in potential fluctuations, which was theorized to be a result of high densities of donors and acceptors^30,33,34^. EBIC has shown both increases and decreases of current collection at GBs^35,36^. Here we also show a semi-statistical chemical study at the nanoscale for 20 GBs by means of APT. The chemistry at GBs will be compared to their adjacent grain interiors, which will explain some of the remarkably diverse reports from many studies of electrical properties at grain boundaries^14,37^.

## Results and Discussion

### Description of Analysis

All data discussed in the subsequent sections are taken from a 20.3%-efficient cell grown on a specialty glass that contains both K and Na^38^. Table 1 summarizes the chemistry and performance. Ga/Ga + In (GGI) for the cell is 0.26, and Cu/Ga + In (CGI) is 0.94.

**Table 1:** Device characteristics of the solar cell used for APT analysis.

Eff (%)	20.3
Jsc (*mA/cm* ^2^)	35.2
FF (%)	80.2
Voc (*mV*)	718

The samples were prepared for APT and TEM analysis using an FEI Helios 600i DualBeam focused ion beam/scanning electron microscope (FIB/SEM) and FEI Nova NanoLab, similar to the technique described in Ref.^39^. APT data were collected using a LEAP 4000X Si instrument manufactured by Cameca Instruments, Inc., with laser energy of 5 pJ, a set point base temperature of 40 K, a detection rate of 1.5 ions per 100 pulses, and a laser pulse rate of 500 kHz. Laser energy and base temperature were optimized to get similar evaporation rates of the constituent elements for an accurate chemical profile of the device that correlates well with X-ray fluorescence (XRF) measurements of high-efficiency CIGS. A Philips CM200 TEM was used to capture the specimen dimensions before APT with the hardware described in Ref.^40^ that allowed for more accurate 3-D reconstructions.

**Figure 1:**
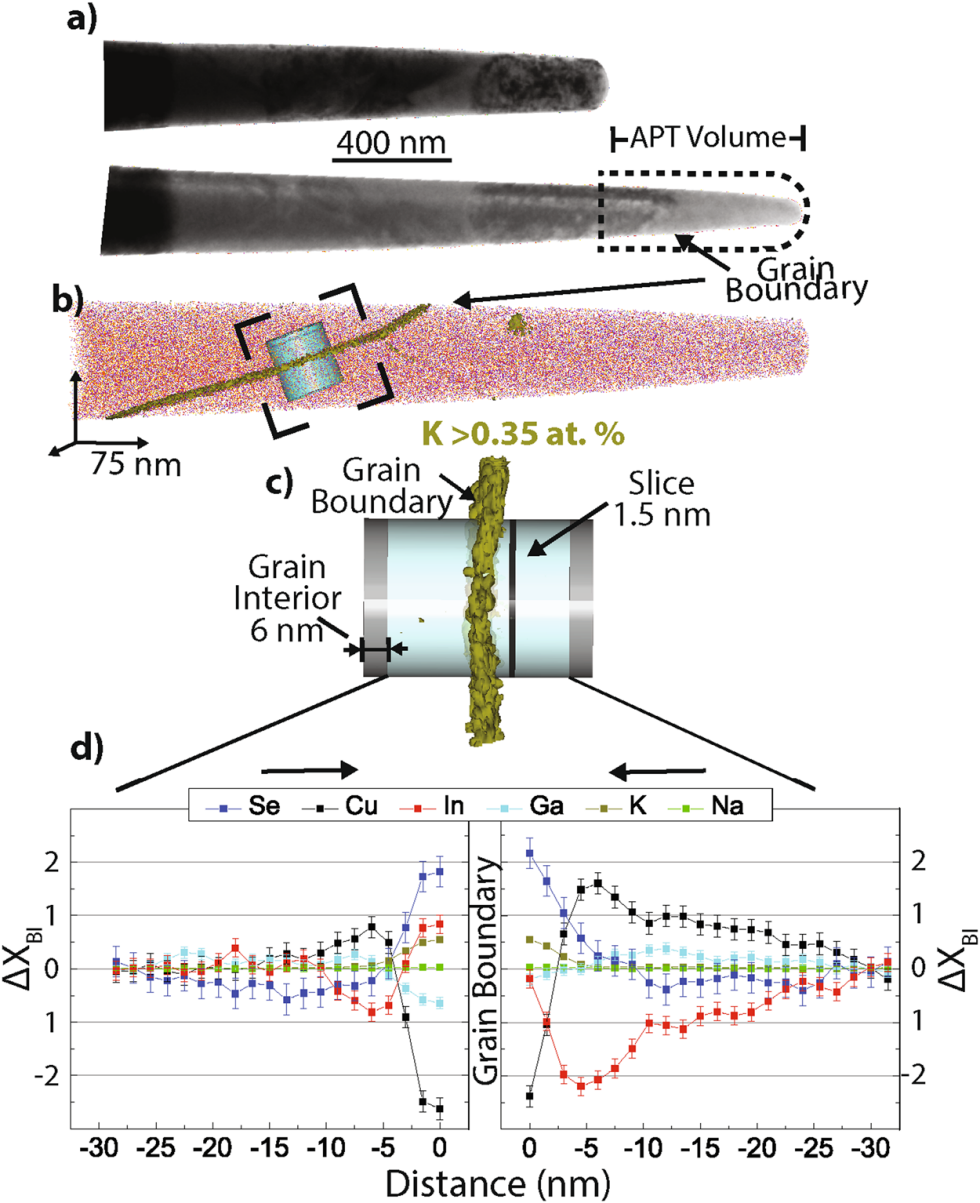
Change in chemistry from the grain boundary to the grain interior ${({\rm{\Delta }}{\rm{{\rm X}}}}_{BI})$. (**a**) Side-by-side of TEM images of the specimen before and after atom probe analysis used to identify volume of APT reconstruction. Top (bottom) is a TEM image taken after (before) atom probe analysis. (**b**) Reconstructed volume capturing a GB. Less than 1% of In (red), Se (blue) and Ga (yellow) ions are shown to give an illustration of the specimen tip shape. An isoconcentration surface of 0.35 at. % K (dark yellow) illustrates segregation to the GB. (**c**) A cylindrical region of interest, taken from dotted square box in (**b**), used to enclose the reconstructed ions for chemical analysis. The grain boundary is determined by K segregation, shown in dark yellow. (**d**) Average composition (at. %) of the first 6 nm (four slices) of both ends of the cylinder was calculated as a grain-interior chemical reference. That value was subtracted from the composition of each slice (1.5 nm thick along the axis of the cylinder), leading to the grain boundary, which resulted in a relative change of chemistry ${({\rm{\Delta }}{\rm{{\rm X}}}}_{BI})$ as a function of distance from the grain boundary. Errors: ± 0.2 at. % Cu, ± 0.1 at. % In, ± 0.3 at. % Se, ± 0.07 at % Ga, 0.04 at. ± % K, and ± 0.03 at. % Na.

Figure 1 demonstrates an example of chemical grain-boundary analysis discussed in subsequent sections. Figure 1(a) shows TEM images taken before and after atom probe analysis. Less than 1% of In (red), Se (blue) and Ga (yellow) ions are shown to give an illustration of the specimen tip shape. The dimensions of the physical volume measured by APT were confirmed by TEM, which was used to improve the accuracy of the reconstructed volume. For all APT specimens discussed in the subsequent sections, a TEM image was taken before, but not always after, atom probe analysis, depending upon whether the specimen survived the atom probe run. An APT reconstruction containing a GB is shown in Fig. 1(b). An isoconcentration surface of 0.35 at. % K (dark yellow) illustrates segregation to the GB in Fig. 1(b,c). Parameters such as compression factor, ion density, efficiency rate, and sphere-to-cone ratios were adjusted, so the GB in the reconstruction and TEM image were correlated. Mass spectra were background corrected and all peaks associated to known ions were ranged at full-width hundredth max. Chemical profiles perpendicular to the GBs were then calculated. Alkali segregation defined where the GB was located. In most cases this was verified by TEM diffraction contrast, and in other cases, the sample was either too thick, or there was a triple-point GB, making the paths difficult to decipher due to the 2-D projection information collected in a TEM image. Only planar Na and/or K segregation qualified as a grain boundary. Intra-grain “rod-like” defects where Na and K also segregated were observed within the analyzed specimens. These types of defects will not be discussed further in this contribution. A good overview of line defects present in Cu(In, Ga)Se_2_ may be found in Ref.^41,42^.

The grain boundaries for which diffraction information using transmission electron microscopy was collected indicated they were random, high-angle grain boundaries. This information was not determined for all of the analyzed grain boundaries, due to the time-consuming nature of such analyses. They are most likely not twin boundaries, because those have been shown to be benign and contain no alkali segregation^32^. Figure 1(c) shows a cylindrical region of interest used to determine a chemical profile perpendicular to the GB. The cylinder is divided into 1.5-nm slices, which enclose many ions (~10 K) used for statistical chemical analysis. The GB bisects the cylinder, and the composition from the grain interior (either end of the cylinder) is compared to the composition at the GB. The average composition (at. %) of the first 6 nm (four slices) from both ends of the cylinder was calculated as a “grain-interior (GI)” chemical reference. That value was then subtracted from the composition of each slice (1.5 nm thick along the central axis of the cylinder) leading to the GB, which resulted in a relative change of chemistry as a function of distance from the GB to the GI. This relative change (at. %) will be defined as ${{\rm{\Delta }}{\rm{{\rm X}}}}_{BI}$, where Χ is the chemical species (Cu, In, Ga, Se, Na, and K) and subscript BI denotes boundary-to-interior. Statistical errors were calculated to be: ± 0.2 at. % Cu, ± 0.1 at. % In, ± 0.3 at. % Se, ± 0.07 at. % Ga, 0.04 at. ± % K, and ± 0.03 at. % Na. Analysis was administered from both sides of the GB, which resulted in two separate ${{\rm{\Delta }}{\rm{{\rm X}}}}_{BI}$ values for each GB. Every GB analyzed was within 1 μm of the p-n junction, verified by a TEM measurement of the distance from the molybdenum-back contact to the reconstructed volume, which was in all cases greater than 1 μm.

### Statistical ${\boldsymbol{\Delta }}{{\boldsymbol{{\rm X}}}}_{{\boldsymbol{BI}}}$

**Figure 2:**
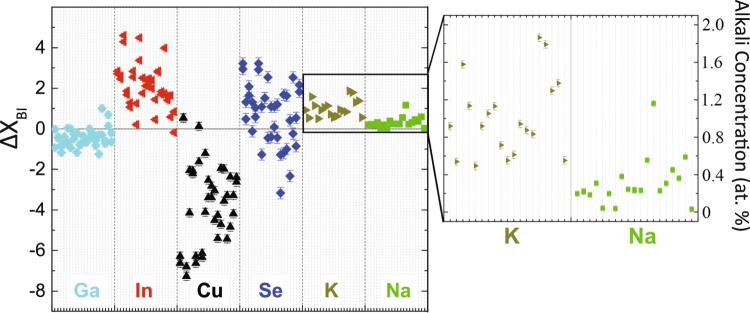
Statistical ${{\rm{\Delta }}{\rm{{\rm X}}}}_{BI}$. ${{\rm{\Delta }}{\rm{{\rm X}}}}_{BI}$ values of 20 grain boundaries were calculated as a relative change in chemistry from the grain boundary to grain interior, as described in Fig. 1. The errors for the different elements are ±0.2 at. % Cu, ±0.1 at. % In, ±0.3 at. % Se, ±0.07 at % Ga, 0.04 at. ±% K, and ±0.03 at. % Na.

Figure 2 shows ${{\rm{\Delta }}{\rm{{\rm X}}}}_{BI}$ for all elements from the 20 grain boundaries and their adjacent grains. Each dash mark at the x-axis (20 for each element) corresponds to a light grey vertical line, which represents the ${{\rm{\Delta }}{\rm{{\rm X}}}}_{BI}$ value for each GB. There are two values for every vertical line, indicating two ${{\rm{\Delta }}{\rm{{\rm X}}}}_{BI}$ values for both grains that “sandwich” a GB. In many cases, the two values are nearly identical; but, in some cases they are notably different, which indicates the chemical variability from grain to adjacent grain. 95% (38/40) of ${{\rm{\Delta }}{\rm{{\rm X}}}}_{BI}$ were Cu-poor and 97.5% (39/40) were (In + Ga)-rich, which is most likely a result of many low-energy-of-formation charge-neutral ordered defect pairs. A negative value of ${{\rm{\Delta }}\text{Cu}}_{BI}$ supports the theory of a decrease in the VBM, resulting in a hole barrier^43–47^. However, there is a wide degree of ${{\rm{\Delta }}\text{Cu}}_{BI}$, leading to a wide degree of potential barrier height. For example, ${{\rm{\Delta }}\text{Cu}}_{BI}$ ranges between 0.5 to −7.5 at. %, ${{\rm{\Delta }}\mathrm{In}}_{BI}$ between −0.3 and +4.7, ${{\rm{\Delta }}\mathrm{Ga}}_{BI}$ between −1.7 and 1 at. %, ${{\rm{\Delta }}\text{Se}}_{BI}$ between −3.14 and +3.14 at. %, ${{\rm{\Delta }}\text{Na}}_{BI}$ between 0.02 and 1.19 at. %, and ${{\rm{\Delta }}K}_{BI}$ between 0.49 and 1.86 at. %. From these data, a good overall picture is presented of the variability of the chemistry at the GBs, along with the extent of impurity atom segregation. As electrons and holes approach the GB, they may experience different outcomes due to the variable local defect density, which will be discussed in the following sections.

### Band Structure

#### Valence- and Conduction-Band Offsets

A well-understood relationship between the ratio of Ga content to the Group III elements and bandgap for Cu(In, Ga)Se_2_ is given by equation (1)^48^. The overall change in bandgap due to Ga incorporation is primarily due to the change in the conduction-band minimum (CBM), whereas Cu content is directly related to a shift in VBM, because of the Cu-d and Se-p anti-bonding hybridized states^15^. A drop in Cu results in a drop in VBM. We have previously shown a linear correlation to the Cu off-stoichiometry to bandgap by equation (2)^49^. The individual contributions of the VBM and the CBM to the total bandgap are given in equations (3) and (4) (see Ref.^49^ for detailed explanation).

1 $${E}_{g}(CIGS)=(1-x){E}_{g}^{CIS}+x{E}_{g}^{CGS}-bx(1-x),$$

2 $${E}_{g}(CIGS)=(1-x){E}_{g}^{CIS}+x{E}_{g}^{CGS}-bx(1-x)+\alpha (25-{X}_{Cu}),$$

3 $$Offse{t}_{VBM}(eV)=(1-\beta )x\{{E}_{g}^{CGS}-{E}_{g}^{CIS}-b(1-x)\}+(1-\sigma )\alpha (25-{X}_{Cu}),$$

4 $$Offse{t}_{CBM}(eV)=\beta x\{{E}_{g}^{CGS}-{E}_{g}^{CIS}-b(1-x)\}+\sigma \alpha (25-{X}_{Cu}),$$

**Figure 3:**
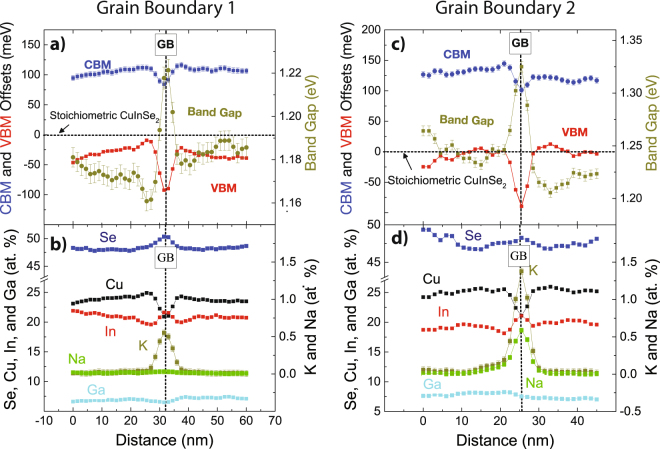
Composition and band profiles from two representative grain boundaries. The errors for the different elements are ±0.2 at.% Cu, ±0.1 at.% In, ±0.3 at.% Se, ±0.07 at% Ga, ±0.04 at. %K, and ±0.03 at.% Na. The maximum errors for the band profiles are ±1.5 meV VBM, ±4.5 meV CBM, and ±5.5 meV band gap.

Composition profiles of two grain boundaries, shown in Fig. 3(b,d), were used to calculate band gap, CBM, and VBM (Fig. 3(a and b)) using equations (2),(3) and (4). The band offsets are calculated with respect to stoichiometric CuInSe_2_ (which is shown in Fig. 3(a) and Fig. 3(c) as the horizontal axis). The total band gap is calculated by adding the VBM and CBM offsets to the known band gap of CuInSe_2_ (1.04 eV). Both GBs have a sharp increase in band gap (~45 and 110 meV over 4.5 nm) due to a reduction in Cu content leading to a sharp drop in the VBM. The CBM is also reduced due to a decrease in GGI for both GBs (−25 and −50 meV for GB 1 and 2, respectively). The potential barrier to charge carriers at a particular GB is related to the difference of chemistry from the GB to grain interior which varies due to chemical heterogeneity of the neighboring grains. The alkali concentration is different for both GBs (~1.4 and 0.5 at. % K, ~0 and 0.5 at % Na for GB1 and 2, respectively) demonstrating a representative range that is discussed below.

**Figure 4:**
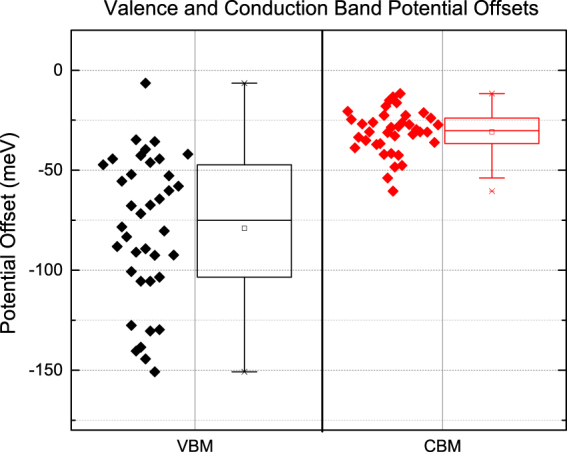
Valence band maximum (VBM) and conduction band minimum (CBM) potential offsets. Calculations were made by ${{\rm{\Delta }}{\rm{{\rm X}}}}_{BI}$ and ${{\rm{\Delta }}\mathrm{GGI}}_{BI}$ values from twenty gain boundaries. Average values: −79 ± 36 meV and −31 ± 11 meV, for VBM and CBM respectively. The right sides indicate averages at the center line of box, while the edges of box are 1 sigma standard deviations and the capped lines indicate 2 sigma.

Composition profiles taken from all 20 GBs in Fig. 2 were used to calculate the CBM and VBM offsets from the GI to the GB. Figure 4 shows a variety of VBM and CBM offsets, due to Cu and Ga content, respectively. The average potential offset for the VBM and CBM is −79 ± 36 meV and −31 ± 11 meV, respectively. Couzine *et al*. reported a GGI reduction at the GB and attributed it to In_Cu_ defect energy being lower than that of Ga_Cu_^50^. This contribution provides a quantitative statistical analysis and helps support their findings. Indium may be more likely to occupy the Cu site than Ga by realizing Pauling’s rules. The ionic radii and coordination number for In are more similar to Cu than Ga to Cu. In addition, APT results show that increases of Cu content lead to increases in Ga content, most likely due to a high energy of formation an antisite defect, Ga_Cu_ (See Fig. 3 for a representative example).

The geometry of the VBM potential barrier resembles a triangle; its width is related to its magnitude, where higher barriers are wider. GB width varied from 3 to 7 ± 1.5 nm, depending on the degree of ${{\rm{\Delta }}{\rm{{\rm X}}}}_{BI}$. Volumes of alkali content greater than 0.2 at. % were picked to determine what constitutes a grain boundary. However, caution must be taken in these measurements, because local distortions most likely exist due to the difference in evaporation from the GI to the GB. A higher atomic density was measured at GBs, indicating a lower evaporation field that results in a reduction of spatial resolution^51^. Both the height and the width of the potential barrier are key parameters for recombination reduction, due to thermo-ionic emission across the barrier and tunneling through the barrier. Taretto *et al*. calculated the transmission probability as a function of height and width of a square barrier^52^. They showed that the barrier must have a minimum height of 300 meV and minimum width of 3 nm to impede recombination for a high-efficiency device. Therefore, a 80-meV VBM barrier at a charge-neutral GB may not be enough for optimal passivation.

#### Alkalis and Band Structure

A well-defined relationship between K and Na and band structure at grain boundaries and grain interiors still needs to be expanded upon. The lowest energy of formation for a K or Na substitutional defect is (K, Na)_Cu_ for CIGS and ${(K,Na)}_{{V}_{Cu}}$ for the ordered vacancy compound of CIGS, which is predicted by hybrid-functional theory^45,53,54^. The possible existence of (K_x_Cu_(1−x)_)(In_y_Ga_(1−y)_)Se_2_ and (Na_x_Cu_(1−x)_)(In_y_Ga_(1−y)_)Se_2_ alloys at the GBs further convolutes the band structure. Because Na and K are both isovalent with Cu and possess no p-orbital, they may mimic that of a V_Cu_ with regards to the VBM. However, this assumption still needs to be explored. Because alkali segregation at the GBs is at most ~2 at. %, its contribution to the band structure may be minimal.

**Figure 5:**
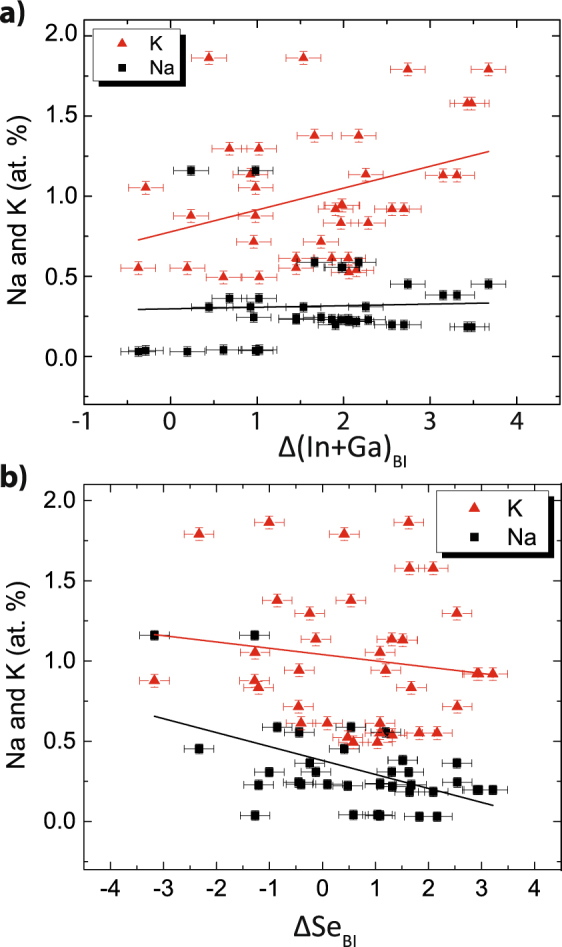
Alkali relationship to n-type defects. (**a**) Alkali segregation is compared to ${\rm{\Delta }}{({\rm{In}}+{\rm{Ga}})}_{BI}$. A positive slope may indicate a correlation of alkali incorporation to a reduction of the n-type defect (In, Ga)_Cu_. However, no clear relationship is measured. (**b**) Alkali incorporation is compared to ${{\rm{\Delta }}\text{Se}}_{BI}$. A negative slope may indicate a correlation of alkali segregation to a reduction of the n-type defect V_Se_. Again, no clear relationship is measured.

If alkalis reside on Cu sites, then their contribution to charge carriers should be benign; yet, their incorporation has long been shown to increase p-type conductivity^55,56^. Long-standing theories that describe the phenomenon relate the presence of alkalis to the reduction of n-type defects: (In, Ga)_Cu_ or V_Se_^26,57–59^. However, there has been little experimental verification. Figures 5(a) and 4(b) plot the alkali content versus ${\rm{\Delta }}{({\rm{In}}+{\rm{Ga}})}_{BI}$ and ${{\rm{\Delta }}\text{Se}}_{BI}$ at grain boundaries, respectively. The black lines in the Figure represent linear fits. An increase in ${\rm{\Delta }}{({\rm{In}}+{\rm{Ga}})}_{BI}$ and a decrease in ${{\rm{\Delta }}\text{Se}}_{BI}$ are presumed to lead to n-type defects: (In, Ga)_Cu_ and V_Se_,. Therefore, a negative slope of ${{\rm{\Delta }}\text{Na}}_{BI}$ and/or ${{\rm{\Delta }}K}_{BI}$ versus ${{\rm{\Delta }}\text{Se}}_{BI}$ would result in a correlation of alkali concentration to a decrease of n-type defects. Similarly, a positive slope of ${{\rm{\Delta }}\text{Na}}_{BI}$ and/or ${{\rm{\Delta }}K}_{BI}$ versus ${\rm{\Delta }}{({\rm{In}}+{\rm{Ga}})}_{BI}$ would also result in a correlation of alkali concentration to an increase of n-type defects. Based on the linear fits, the slopes are opposite in sign and no such correlations are apparent. Furthermore, the inclusion of Na and K may result in the opposite—an overall n-type doping if they occupy what was a V_Cu_ defect from a charge-neutral ordered defect pair.

The work of Yan *et al*. reported KPFM measurements on polycrystalline CIGS grown with and without alkalis. An increase in the work function (indicative of n-type doping) at GBs was reported for the absorber grown with Na, yet there was no measurable change work function at GBs for the absorber grown without alkalis^60^. With Yan *et al*.’s work and no correlation made of alkali incorporation to a reduction of n-type defects (Fig. 5), we propose that alkalis may act as an n-type dopant at GBs. Yuan *et al*. recently predicted that the diffusion of alkalis at the GIs is the reason why alkalis increase p-type conductivity^55^. They show that the solubility limit of Na and K at GIs is increased with temperature, resulting in larger quantities of Na_Cu_ (K_Cu_) during growth. When the absorber cools, Na and K out-diffuse, leaving a high density of V_cu_’s. The alkalis are then rinsed away before the CdS chemical bath. We now add to this theory and provide a missing link between the seemingly contradictory roles of alkalis at GBs and enhanced p-type doping. With the addition of the GB data presented in this report and in conjunction with the theory presented by Yuan *et al*., we presume alkalis increase the p-type doping at GIs and become n-type dopants at GBs. Equation (5) is a point defect bottom which combines Yuan *et al*.’s theory for bulk material with the present contribution’s predicted grain boundary point defects (Na and K may be used interchangeably in equation). In other words, ${(Na,K)}_{Cu}$ out-diffuse from the GI, leaving a V_Cu_ behind which may either be rinsed away at the surface or occupy a V_Cu_ at the GB, leading to a p-type doping at the GI and an n-type doping at the GB. If the grains are columnar, the band bending at the GB induced by the n-type doping would reduce recombination and increase charge collection. Furthermore, the variable chemistry at the GB leads to variable magnitudes of band bending and potential barriers and may help explain the diverse electrical properties exhibited by techniques such as EBIC, KPFM, and CL (see section 4). The benefit of alkali incorporation may be two fold: increase p-type conductivity, and increase band bending at the GB.

5 $$\begin{array}{c}({\rm N}{\alpha }_{x}C{u}_{1-x})(I{n}_{x}G{a}_{1-x})S{e}_{{2}_{bulk}^{0}}+2{V}_{C{u}_{GB}^{{\prime} }}+I{n}_{C{u}_{GB}^{\circ\circ}}+2{h}_{GB}^{\circ }+2{e}_{GB}^{\text{'}}\to \\ \quad Cu(I{n}_{x}G{a}_{1-x})S{e}_{{2}_{bulk}^{0}}+{V}_{C{u}_{bulk}^{{\prime} }}+{h}_{bulk}^{\circ }+N{a}_{Cu\,GB}^{\circ }\\ \quad +I{n}_{C{u}_{GB}^{\circ\circ}}+{V}_{C{u}_{GB}^{{\prime} }}+{h}_{GB}^{\circ }+2{e}_{GB}^{\text{'}}\end{array}$$

#### Fluctuating potentials and high degree of compensation

Cu(In, Ga)Se_2_, a highly compensated semiconductor, has many more donors and acceptors (~10^19^) than free charge carriers (holes ~10^17^)^45^. At high enough temperatures where the donors and acceptors are ionized, the net free carrier concentration is the difference between the number of donors and acceptors shown in equation (6), where N_D_ is donor concentration, N_A_ is acceptor concentration and N_t_ is total free carrier concentration.

6 $${N}_{t}={N}_{D}+{N}_{A},$$

This leaves many ionized defects, whose electric fields cause fluctuating potentials on charge carriers. Shklovskii and Efros^61^ and Dirnstorfer *et al*.^62^ defined the relationship between the average potential fluctuations and ionized defects for highly doped semiconductors and Cu(In, Ga)Se_2_, respectively. From Fig. 1, we predict that the bands will fluctuate appreciably as a function of proximity to the GB, due to the increasingly large amount of compensation, i.e., many n-type point defects (In_Cu_) and many p-type defects (V_Cu_). The average magnitude of potential fluctuations is given by equation (7), where the defect density is assumed to be evenly distributed^61,62^:

7 $$\gamma (R)=\frac{{e}^{2}}{(4\pi \varepsilon {\varepsilon }_{0})}\frac{{({N}_{t}{R}^{3})}^{1/2}}{R},$$

where R is the radius of the enclosed volume and N_t_ is the total density of charged ions. Charge carriers will screen the ions and only be affected by ions within a radius, r_s_ related to charge-carrier density, N_t_ defined by equation (8), where p is the hole density:

8 $${r}_{s}=\frac{{N}_{t}^{1/3}}{{p}^{2/3}},$$

Combining equations (7) and (8) gives the potential fluctuation in terms of N_t_ and charge carrier density (ρ):

9 $$\gamma ({r}_{S})=\frac{{e}^{2}}{(4\pi \varepsilon {\varepsilon }_{0})}\frac{{{N}_{t}}^{2/3}}{{p}^{1/3}},$$

Activation energies of defects will also be affected by the potential fluctuations. Podor *et al*. showed that the energy to ionize a defect decreases as a function of the magnitude of potential fluctuations as defined by equation (10)^63^:

10 $${E}_{A}={E}_{o}-\alpha {N}_{t}^{1/3},$$

where *E*_*A*_ is the activation energy, *E*_*o*_ is the thermal ionization energy, α is a constant approximately 4 × 10^−5^ meV, and Nt is the defect density. Based on Fig. 1, the degree of compensation at the GB may be extreme, where a high density of V_Cu_ and (In, Ga)_Cu_ defects (Nt ≈ 10^21^) are present. In this case, donors and acceptors would be at the band edges. Further fundamental analysis is needed to relate the increase in potential fluctuations at the GBs to overall cell performance.

**Figure 6:**
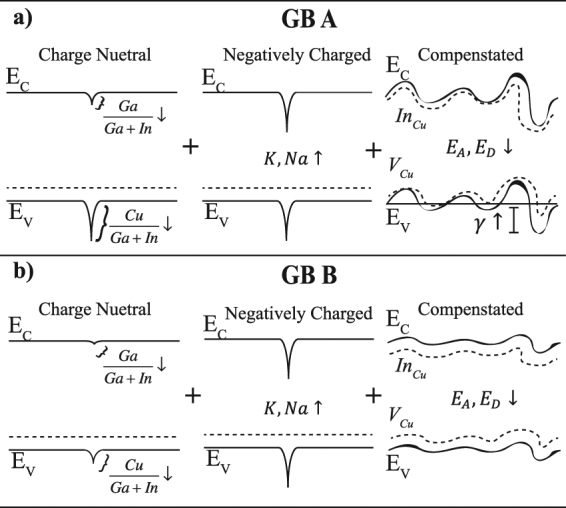
Grain-boundary band profiles. Grain-boundary A (B) refers to a large (small) ${{\rm{\Delta }}X}_{BI}$. Grain-boundary A has a larger potential barrier to holes at the valence-band minimum. All grain boundaries are presumed to be doped n-type from alkali incorporation, where both have a positive potential. Grain-boundary A also exhibits larger potential fluctuations due to increased compensation. Dashed lines in the “Charge Neutral” and “Negatively Charged” diagrams represent the Fermi energy, and the position of the defect states, V_Cu_ and In_Cu_, in the “Compensated” diagram. E_A_ and E_D_ are activation energy of acceptors (V_Cu_) and donors (In_Cu_) respectively.

Figure 6 provides a summary of our findings, demonstrating the band structure for two types of grain boundaries with small and large ${{\rm{\Delta }}X}_{BI}$. Most grain boundaries measured are Cu- and Ga-poor and In-rich, resulting in negative potential shifts of the VBM and CBM, though not necessarily to the same extent. All random, high-angle grain boundaries analyzed had alkali segregation, which is presumed to lead to n-type doping and result in a positive potential.

#### Understanding EBIC, CL, and KPFM trends

We have shown a complex band structure resulting from the variable chemistry discussed in the preceding sections. We now discuss possible reasons why electrical properties measured by EBIC are also variable. EBIC is taken at short-circuit conditions and contrast is determined by current collection. Light (dark) regions, which correlate to high (low) current collections, could result from increased (decreased) charge separation and lead to a high (low) probability of carriers reaching the contacts. A GB with large n-type doping (due to significant K and Na content) would lead to a large potential and efficient charge collection. A GB with small potential could lead to inefficient charge separation and a large density of recombination centers. Also, a charge-neutral GB that contains many compensating defects, resulting in an offset at the VBM in conjunction with a potential due to n-type doping, would lead to even greater efficiency in charge separation. However, the electron beam would generate fewer electron-hole pairs, due to a larger bandgap. Clearly, there is a wide variety of band profiles that lead to variable current collection at GBs. Many works report a red shift at GBs measured by CL. The degree of red shift will be highly variable because the donor-acceptor pair transitions depend on V_Cu_ and (In, Ga)_Cu_ defect densities. As shown in Fig. 2, the defect densities vary by more than an order of magnitude. The potential fluctuations also vary according to defect densities; therefore, they would lead to variable degrees in red shifts, because the donor and acceptor activation energies will also vary (equations (6),(7), and (10)). A wide variety of GB potentials are shown in many KPFM studies. A possible explanation is the variable alkali concentration, with the alkali presumed to be an n-type dopant. Because the alkali concentration also varies (Fig. 2), the potential should also vary.

## Conclusion

The relationship between relative chemical changes from the grain interiors to grain boundaries ${\rm{\Delta }}{({\rm{X}})}_{BI}$ relate and the band structure of CIGS solar cells was explored for a large number of grain boundaries. Highly accurate chemical profiles show large chemical variability, which lead to a spectra of band profiles. We provide the following five main conclusions: 1) Grain-boundary chemistry is highly variable, resulting in a wide distribution of potential barriers at the VBM (−10 to −160 meV) and CBM (−20 to −70 meV); 2) Na and K segregation is not correlated to hampering donors: (In, Ga)_Cu_ and V_Se_; 3) Na and K are predicted to be n-type dopants at grain boundaries; 4) Potential fluctuations increase as a function of proximity to the grain boundary, due to increases in compensation, which leads to reductions in donor and acceptor activation energies; and 5) The previous four conclusions provide an explanation for electrical property variations seen in EBIC, CL, and KPFM.

## Methods

### Growth Process

CIGS solar cells were prepared on high-temperature specialty glass coated with sputtered molybdenum to serve as a back contact to the device. The CIGS absorber layer was deposited via a three-stage co-evaporation process described elsewhere, with modifications to controllably change the bandgap (Ga) profile^64–66^. The substrate temperature during the second stage, ~615 °C, was measured with a thermocouple placed on the backside of the glass. The devices were then allowed to cool and removed from the vacuum chamber. All absorbers were then finished into devices using the standard CdS and ZnO bilayer described by Contreras *et al*.^64^.

### Sample Preparation

The samples were prepared for atom probe tomography (APT) and transmission electron microscopy (TEM) analysis using an FEI Helios 600i DualBeam focused ion beam/scanning electron microscope (FIB/SEM). A final 2-kV cleaning step was used to reduce the damage to about the outer 2 nm of the sample. This was verified by isolating the Ga regions that have mono-isotopic 69 Ga used in the FIB source from the regions with the naturally occurring 69 Ga and 71 Ga isotopic ratios within APT reconstructions. The end radii of the specimens analyzed ranged from 50–100 nm. APT data were collected using a LEAP 4000X Si instrument manufactured by Cameca Instruments, Inc. We used a laser energy of 5 pJ at a set point temperature of 40 K with a detection rate of 1.5 ions per pulse and a laser pulse rate of 500 kHz. The laser energy and base temperature were optimized to obtain equal evaporation rates of the constituent elements for an accurate chemistry profile of the sample that well represents the stoichiometry of CIGS. A Philips CM200 TEM was used to capture the specimens’ dimensions before and after APT using the hardware described in Gorman *et al*.^40,67^. This procedure allowed for more accurate 3-D reconstructions.

The datasets generated during and/or analysed during the current study are available from the corresponding author on reasonable request^67^.
